# Spinoculation Enhances HBV Infection in NTCP-Reconstituted Hepatocytes

**DOI:** 10.1371/journal.pone.0129889

**Published:** 2015-06-12

**Authors:** Ran Yan, Yongmei Zhang, Dawei Cai, Yuanjie Liu, Andrea Cuconati, Haitao Guo

**Affiliations:** 1 Department of Microbiology and Immunology, Indiana University School of Medicine, 635 Barnhill Drive, Indianapolis, Indiana, 46202, United States of America; 2 Department of Infectious Diseases, Huashan Hospital, Fudan University, 12 Wulumuqi Zhong Rd, Shanghai, 200040, China; 3 Baruch S. Blumberg Institute, Hepatitis B Foundation, 3805 Old Easton Rd, Doylestown, Pennsylvania, 18902, United States of America; Academia Sinica, TAIWAN

## Abstract

Hepatitis B virus (HBV) infection and its sequelae remain a major public health burden, but both HBV basic research and the development of antiviral therapeutics have been hindered by the lack of an efficient in vitro infection system. Recently, sodium taurocholate cotransporting polypeptide (NTCP) has been identified as the HBV receptor. We herein report that we established a NTCP-complemented HepG2 cell line (HepG2-NTCP12) that supports HBV infection, albeit at a low infectivity level following the reported infection procedures. In our attempts to optimize the infection conditions, we found that the centrifugation of HepG2-NTCP12 cells during HBV inoculation (termed “spinoculation”) significantly enhanced the virus infectivity. Moreover, the infection level gradually increased with accelerated speed of spinoculation up to 1,000g tested. However, the enhancement of HBV infection was not significantly dependent upon the duration of centrifugation. Furthermore, covalently closed circular (ccc) DNA was detected in infected cells under optimized infection condition by conventional Southern blot, suggesting a successful establishment of HBV infection after spinoculation. Finally, the parental HepG2 cells remained uninfected under HBV spinoculation, and HBV entry inhibitors targeting NTCP blocked HBV infection when cells were spinoculated, suggesting the authentic virus entry mechanism is unaltered under centrifugal inoculation. Our data suggest that spinoculation could serve as a standard protocol for enhancing the efficiency of HBV infection in vitro.

## Introduction

Hepatitis B virus (HBV) is a hepatotropic enveloped DNA virus that causes transient and chronic hepatitis B in humans [1]. HBV mainly infects hepatocytes and establishes a pool of a nuclear episomal covalently closed circular (ccc) DNA form of the viral genome, which serves as transcription template for all the viral RNAs, including 3.5kb precore mRNA and pregenomic (pg) RNA, 2.4kb and 2.1kb surface protein mRNAs, and 0.7kb X mRNA. The DNA replication of HBV is catalyzed by viral DNA polymerase in cytoplasmic viral capsid through reverse transcription of the HBV pgRNA precursor, and subsequently the mature double stranded DNA-containing nucleocapsid is enveloped by viral surface glycoproteins and secreted as progeny virion [1, 2].

HBV has infected approximately 2 billion people worldwide, resulting in 350–400 million chronic infections; this significant epidemic level of hepatitis B is partly due to the high infectivity of HBV *in vivo* when the prophylactic vaccination is not in place [3]. It has been reported that the inoculation of a single HBV infectious particle in chimpanzee was able to establish an acute HBV infection in 100% of the hepatocytes [4]. Ironically, a robust HBV *in vitro* infection has been difficult to achieve in the hepatocyte-derived cells, which is, presumably, due to the loss of HBV receptor(s) in the transformed or cancerous hepatocytes, or the destruction of *in vivo* architecture and/or environment of hepatocytes when the cells are plated into monolayer on petri dish. Although primary human hepatocytes (PHHs) and the HepaRG cell line can be used for certain HBV infection experiments, the PHHs are costly with limited supply, and their genetic background and susceptibility to HBV infection vary from donor to donor [5]; in these regards, the HepaRG system does have advantages over PHH but time-consuming cell proliferation and differentiation steps are required prior to infection [6]. What’s more, the HBV infectivity in these two cell systems remains extremely inefficient. Thus, the basic and antiviral research of HBV in the context of a complete viral life cycle have been hampered for a long period of time. Recently, the Na^+^-taurocholate cotransporting polypeptide (NTCP) has been identified as a functional HBV receptor, creating a paradigm-shifting platform for HBV research [7]. Now, the reconstitution of NTCP expression in commonly used hepatocyte-derived cells (i.e. HepG2 and Huh7) confers permissiveness of cells to HBV infection, fostering novel mechanistic and therapeutic studies on the early steps of the HBV life cycle, including receptor-mediated HBV entry, uncoating, and first round cccDNA formation, etc. Nevertheless, the reported HBV infectivity of NTCP-expressing cells varies among different laboratories under different infection conditions, but the average percentage of HBcAg or HBsAg positive cells remains low as revealed by immunofluorescence [7–11]. Therefore, further optimization and standardization of the protocol for HBV infection in NTCP-expressing cells is warranted.

Spinoculation, also known as centrifugal inoculation, has been widely used to augment the *in vitro* infectivity of a variety of viruses in adhesion and suspension cultures since its first application in virus infection in 1960’s [12, 13]. Various mechanisms for this enhancement have been proposed, from ultrastructural changes in the host cell that render it more permissive to viruses [14], to (surprisingly for such low centrifugation speeds) increased deposition of virions on the cell surface [15]. Herein, we established a HepG2-based NTCP-expressing cell line which is permissive to HBV infection, and developed a spinoculation method to enhance HBV infection with the optimized cell culture conditions and viral inoculum size. In addition, the enhanced HBV infection by spinoculation is in an NTCP-dependent manner, suggesting that spinoculation promotes HBV infection through the authentic viral entry mechanism. Therefore, spinoculation could serve as a routine procedure in the protocol of *in vitro* HBV infection.

## Materials and Methods

### Plasmids and drugs

Plasmid pcDNA6-NTCP that expresses the rhodopsin C9-tagged human NTCP and the blasticidin resistance gene in mammalian cells was kindly provided by Dr. Wenhui Li (National Institute of Biological Sciences, Beijing, China) [7]. The 1.3-mer HBV plasmid pHBV1.3 has been described previously and was further modified to remove an additional polyadenylation signal in the vector sequence downstream of the HBV genome, by which prevents the synthesis of overlength HBV mRNAs with 3’ non-HBV sequence [16]. HBV entry inhibitor Myrcludex-B is a gift from Dr. Stephan Urban (University Hospital Heidelberg, Heidelberg, Germany) [9]. Cyclosporin A and Sodium taurocholate were purchased from Sigma-Aldrich (St. Louis, MO).

### Establishment of cell line that stably expresses NTCP

The HepG2 cells (Clone N6, a gift from Dr. David Anderson (Burnet Institute, Melbourne, Australia)) were maintained in Dulbecco’s modified Eagle’s medium (DMEM)-F12 medium (Mediatech, Manassas, VA) supplemented with 10% fetal bovine serum, 100 IU/ml penicillin and 100 μg/ml streptomycin. No endogenous NTCP was detected in HepG2 cells [7, 9] (data not shown). To reconstitute NTCP expression, HepG2 cells were transfected with plasmid pcDNA6-NTCP by Lipofectamine 2000 (Life Technologies) for 48 h, then the transfected cells were split (1:24) and selected with 8 μg/ml blasticidin. Twenty four blasticidin-resistant colonies were picked and expanded into cell lines. The expression level of NTCP in the candidate cell clones were measured by Western blot with antibody against C9-epitope (Santa Cruz Biotechnology, cat#: SC-57432). One cell line that supported high levels of NTCP expression and HBV infection was designated as HepG2-NTCP12 and was used in this study. HepG2-NTCP12 cell line was maintained in the same way as HepG2 cells except for the addition of blasticidin (8 μg/ml).

### Preparation of HBV inoculum

HBV particles were collected from the supernatant of HepDE19 cells as described previously with modifications [17]. Briefly, HepDE19 cells were cultured in tetracycline-free medium to induce HBV replication and virion production, supernatant was collected every other day with culture medium replenishment for 18 days. The pooled supernatant was mixed with polyethylene glycol (PEG)-8000 powder (final concentration of 10%) and gently rotated at 4°C for overnight, HBV particles were then precipitated by centrifugation at 1,000×g for 30 min at 4°C and re-dissolved in serum-free DMEM/F12 medium with 1% volume of the original supernatant samples. The concentrated virus stocks were aliquoted and stored at -80°C.

### HBV particle gel assay and viral RNA/DNA analysis

HBV particle gel assay was performed as previously described [17]. Briefly, the viral particles in the PEG-concentrated HBV inoculum were fractionated by electrophoresis (22 V, 16 h) through nondenaturing 1% agarose gel and capillarilly transferred onto the nitrocellulose filter (Whatman). The DNA-containing viral particles on the membrane were first denatured in a solution containing 0.5 M NaOH and 1.5 M NaCl and then neutralized by 1 M Tris-HCl (pH 7.4) and 1.5 M NaCl, followed by UV crosslink. HBV DNA was detected by hybridization with a [α-32P] UTP (800 Ci/mmol; Perkin Elmer)-labeled minus-strand-specific full-length HBV riboprobe.

Intracellular HBV RNA Northern blot analysis, extracellular and intracellular HBV core DNA and cccDNA Southern blot analysis, were performed according to our previous publications [16, 18–20]. The probe used in Northern blot is an [α-32P] UTP (800Ci/mmol; Perkin Elmer)-labeled plus-strand-specific full-length HBV riboprobe.

The quantification of HBV total DNA was done by using Faststart Essential DNA Probes Master (Roche). The primer and probe sequences used in real-time PCR reaction were: forward primer: 5’-CCGTCTGTGCCTTCTCATCTG-3’, reverse primer: 5’-AGTCCAAGAGTYCTCTTATGYAAGACCTT-3’, and probe: 5’-FAM-CCGTGTGCACTTCGCTTCACCTCTGC-TAMRA-3’. The amplification setting was 0.8 μM of primers and 0.2 μM probe, annealing and extension at 64°C for 45 cycles.

### HBV Infection and spinoculation

HepG2-NTCP12 cells were seeded into collagen-coated 24-well plates with density of 2×10^5^ cells/well and cultured in regular DMEM/F12 medium for overnight, and then the culture medium was switched to primary hepatocytes maintenance medium (PMM), specifically Williams E medium supplemented with 10% FBS, 5 μg/ml transferrin, 10 ng/ml hEGF, 3 μg/ml insulin, 2 mM L-glutamine, 18 μg/ml hydrocortisone, 40 ng/ml dexamethasone, 5 ng/ml sodium selenite, 2% dimethyl sulfoxide (DMSO, cell culture grade), 100 U/ml penicillin, and 100 μg/ml streptomycin [7]. After 24 h, the cells were incubated with HBV inoculum diluted in PMM containing 4% PEG-8000. To perform spinoculation, the HBV inoculated plate was immediately centrifuged at room temperature with specified speed and time. The cells received regular HBV inoculation or spinoculation were transferred to 37°C CO_2_ incubator. 18 h later, the HBV inocula were removed and the infected cells were maintained in regular PMM without PEG before harvest.

### Western blot

Cells in one well of a 24-well plate were lysed in 100 μl of 1× Laemmli buffer and denatured at 95°C for 10min. 25 μl of the cell lysate was resolved in a 12% SDS-PAGE gel, and proteins were transferred onto an Immobilon PVDF-FL membrane (Millipore). The membranes were blocked with WesternBreeze Blocker and probed with antibodies against C9-tag (Santa Cruz Biotechnology, cat#: SC-57432) and β-actin (Millipore, cat# MAB1501). Bound antibodies were revealed by IRDye secondary antibodies and visualized using the LI-COR Odyssey system.

### Immunofluorescence assay

Cells were fixed with 4% paraformaldehyde for 20 min and permeabilized by 0.5% Triton X-100 in PBS for 60 min at room temperature. Cells were subsequently blocked with 10% FBS plus 2% BSA for 60 min at room temperature, and then incubated with anti-C9 tag antibody or anti-HBcAg (Dako, cat#: B0586) diluted in PBS containing 10% FBS and 2% BSA for 2 h at room temperature. After being washed with PBS, the cells were stained with Alexa Fluor 594 dye-conjugated secondary antibody (Life Technologies) and the nuclei were counterstained with DAPI for 60 min at room temperature. Finally, the cells were washed with PBS and then subjected to Nikon Eclipse TE 2000-U fluorescent microscopy or Olympus FV1000 MPE confocal microscopy analysis with the 60× objective. Images were analyzed using the FV10-ASW 3.0 Viewer Software.

## Results

### Establishment of NTCP-reconstituted HepG2 cells

**Fig 1:**
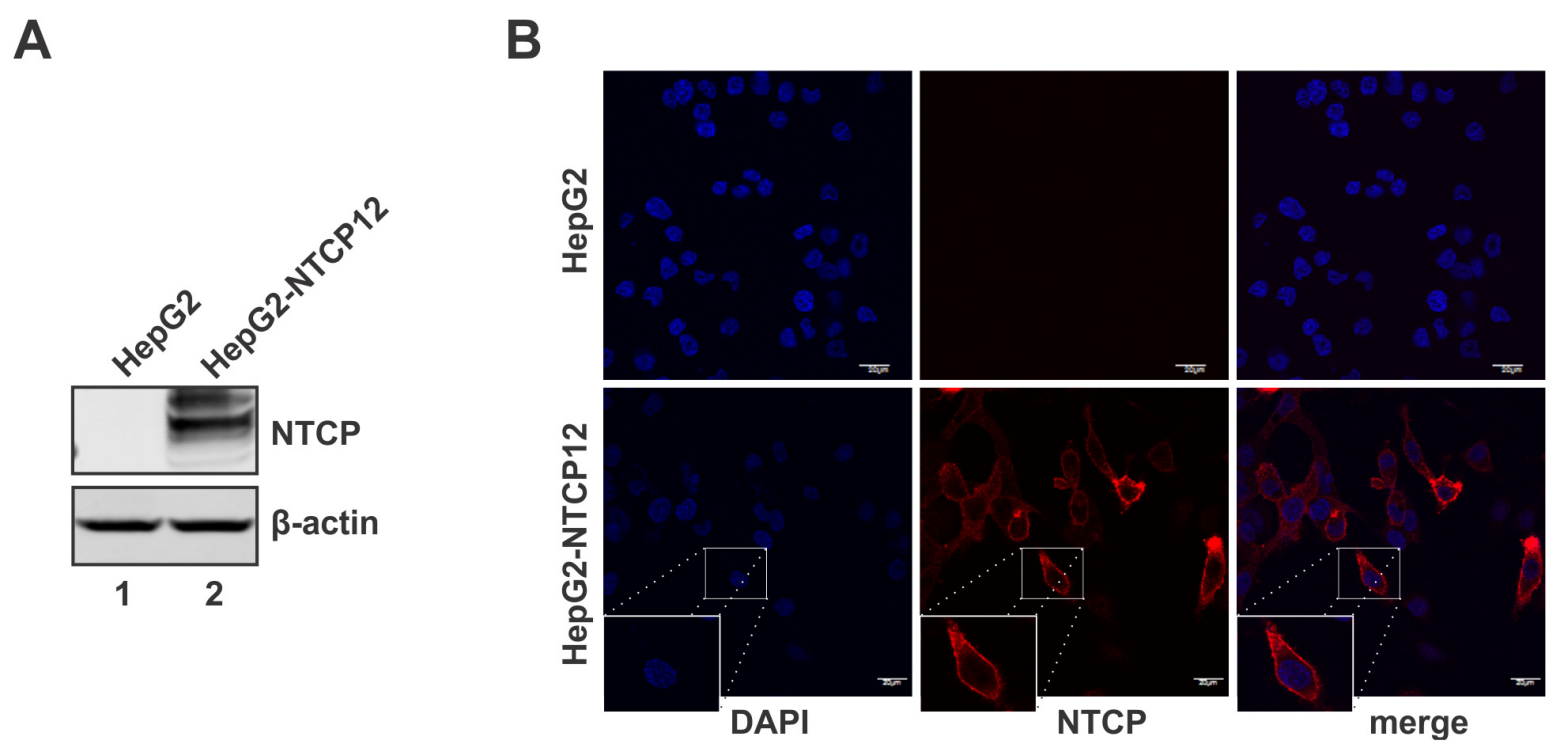
NTCP expression in HepG2-NTCP12 cells. The stable expression of C9-tagged NTCP in HepG2-NTCP12 cells were detected by Western blot (A) and immunofluorescence microscopy (B) using antibody against C9 epitope. The parental HepG2 cells served as negative control.

Based on the previously published literature [7], we transfected HepG2 cells with plasmid pcDNA6-NTCP which expresses the rhodopsin C9-tagged human NTCP and the blasticidin resistance gene, and obtained 16 antibiotic-resistant, NTCP-expressing cell lines. Among them, one cell line that expressed the highest level of NTCP was designated as HepG2-NTCP12 and used in subsequent studies. As shown in Fig 1, the expression of C9-tagged NTCP in HepG2-NTCP12 cells, but not in the parental HepG2 cells, was confirmed by both Western blot and immunofluorescence microscopy analyses. Microscopic imaging demonstrated a marked pattern of cell surface expression of NTCP in HepG2-NTCP12 cells, which is consistent with the localization of endogenous NTCP in hepatocytes [21]. In addition, transfection of plasmid pHBV1.3 into HepG2-NTCP12 cells resulted in HBV RNA transcription and core DNA replication (data not shown), indicating that HepG2-NTCP12 cell line is capable of supporting HBV replication in viral infection, if the early entry steps up to cccDNA formation could be fulfilled.

### Determination of virion genome equivalent in the HBV inoculum

**Fig 2:**
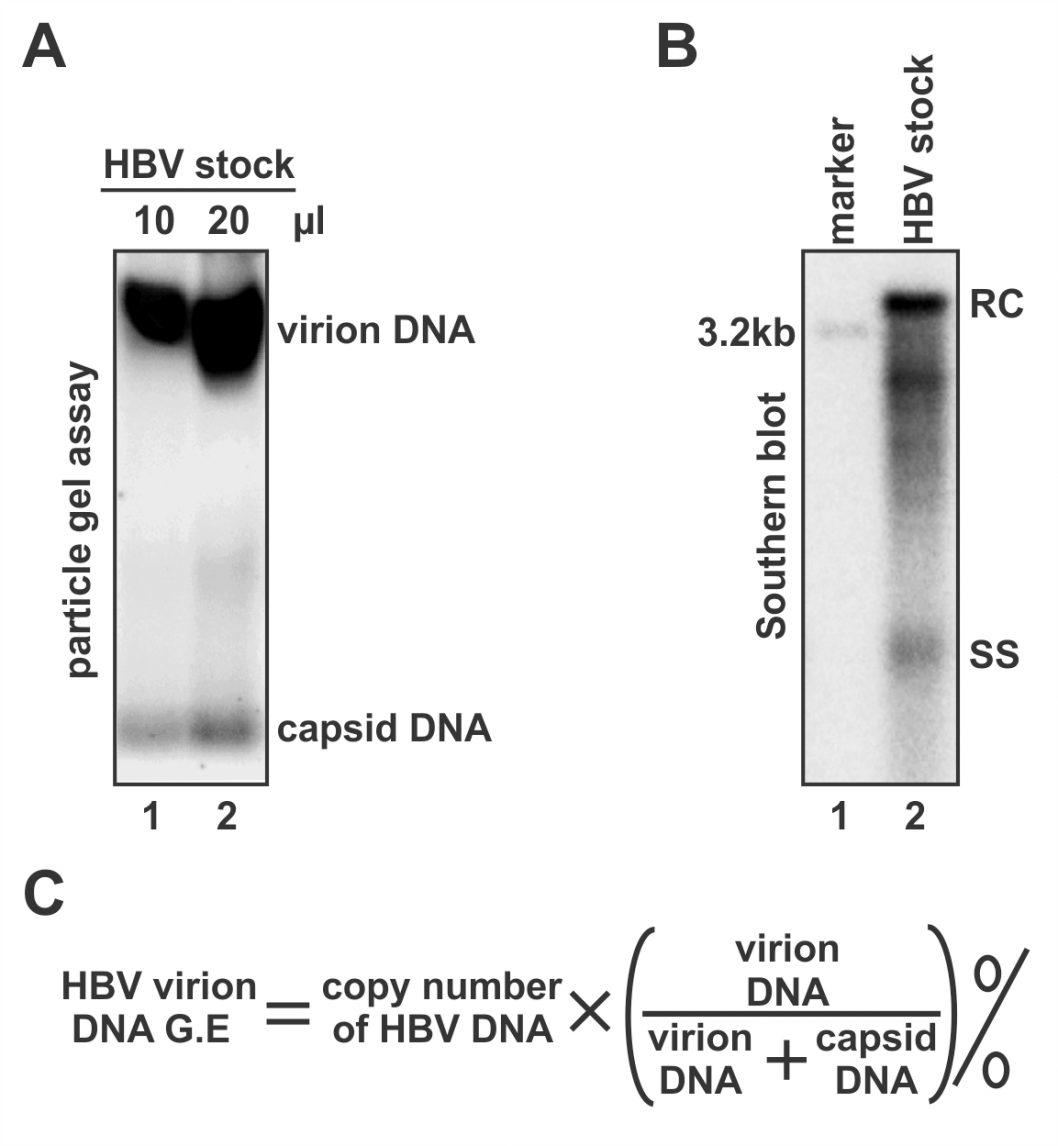
Quantification of the genome equivalent of HBV virion DNA in concentrated HBV particles. (A) HBV virions and naked capsids in the indicated volume of virus stock were separated by native agarose gel electrophoresis, and viral DNA was detected by hybridization. The virion DNA to capsid DNA ratio was calculated by ImageQuant IQTL software using the hybridization signal intensity. (B) HBV DNA were extracted from 10 μl of virus stock and subjected to Southern blot analysis, 100 pg of 3.2 kb HBV linear DNA (approximately 3.1×10^7^ HBV DNA copies quantified by qPCR) served as loading marker. HBV DNA replicative intermediates, including relaxed circular (RC) DNA and single stranded (SS) DNA were labeled. The copy number of total HBV DNA was quantified by using the HBV DNA loading marker as standard. (C) Formula for calculation of HBV virion DNA genome equivalent (v.g.e).

The HBV inoculum was prepared by 100-fold concentration of the virus-containing supernatant from HepDE19 cells with PEG as described previously [6]. It is known that the HBV producing cells secrete enveloped virions and a considerable amount of nonenveloped “naked” capsids in vitro, both contain viral DNA, but only virions being infectious [17, 20, 22, 23]. Furthermore, while HBV virion contains double stranded DNA genome only, the naked capsids contain heterogeneous core DNA replicative intermediates (Cai and Guo, unpublished data). In order to calculate the virion genome equivalent (vge) of the HBV inoculum more accurately, we developed a method to serve this purpose. First, native particle gel assay was performed to determine the ratio between HBV virion DNA and naked capsid DNA in the HBV stock (Fig 2A), then the total amount of particle-associated HBV DNA was quantified by Southern blot hybridization using an HBV double stranded linear DNA with known copy number as standard (Fig 2B). Alternatively, total HBV DNA was quantitated by qPCR (data not shown). Finally, the vge of HBV inoculum was calculated by normalizing the HBV DNA copy number with the percentage of virion DNA among total viral DNA from HBV particles (Fig 2C). By using the above virus titration method, the vge of the HBV inoculum we prepared from HepDE19 cells is approximately 7.6×10^7^ virions/μl.

### Effects of DMSO and inoculum size on HBV infection

**Fig 3:**
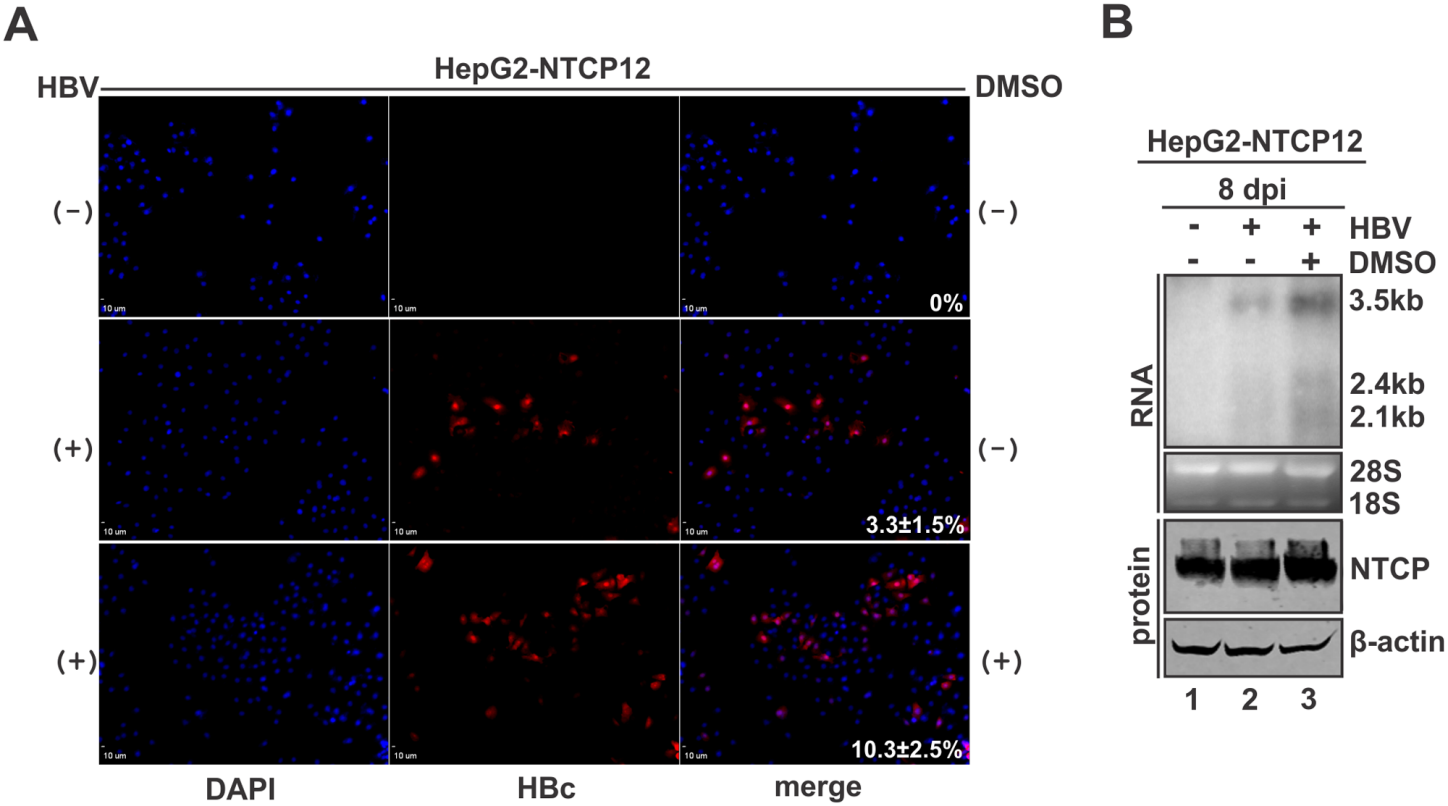
DMSO enhances HBV infection in HepG2-NTCP12 cells. Approximately 5×10^5^ HepG2-NTCP12 cells were mock infected or infected with HBV (100 g.e/cell) in the absence or presence of 2% DMSO. DMSO was added to the PMM medium 24 h prior to the infection and remained present in the entire culture period until the cells were harvested. (A) Cells were immunostained with antibodies against HBV capsid protein (HBcAg) and visualized under fluorescence microscopy. Nuclei were counterstained with DAPI. The image shown represents five different microscopic fields, the percentage of HBcAg-positive cells was indicated (Mean ± SD) (similarly hereinafter). (B) HBV RNA were extracted from the infected cells and analyzed by Northern blot hybridization by using an [α-32P] UTP-labeled plus-strand-specific full-length HBV riboprobe. HBV 3.5 kb precore/pregenomic RNA and the 2.4/ 2.1 kb subgenomic RNA are labeled. Cellular 28S and 18S ribosomal RNA served as loading control. C9-tagged NTCP expression was detected by Western blot with β-actin serving as loading control.

Next, we tested the susceptibility of HepG2-NTCP12 cells to HBV infection. As shown in Fig 3, inoculation of HepG2-NTCP12 cells by HBV at 100 vge/cell exhibited HBcAg immunofluorescent signals in a small percentage of cells at day 8 post infection, but the percentage of HBcAg positive cells was increased by 3 fold to above 10% when the infection was performed in the presence of 2% DMSO in the culture medium (Fig 3A). HBV RNAs were detected in the infected cells by Northern blot, indicating successful mRNA transcription from the established cccDNA during infection. Similar to the enhanced HBcAg expression by DMSO, the compound also increased the steady state levels of HBV RNA in the infected cells (Fig 3B). DMSO has been used as a differentiation inducer for HepaRG cells to become susceptible to HBV infection, partly through upregulating hepatocyte-specific host factors including NTCP and certain hepatic transcription factors required for HBV entry and cccDNA transcription [6, 24]. Consistent with our result, DMSO was also found to enhance HBV infection in NTCP-reconstituted HepG2 and Huh7 cells [9]. Therefore, DMSO was routinely included in the PMM infection medium in following studies. However, viral DNA replicative intermediates were only detected by qPCR, but not by conventional Southern blot (data not shown), indicating that the level of HBV infection and/or replication remained low under this infection condition.

**Fig 4:**
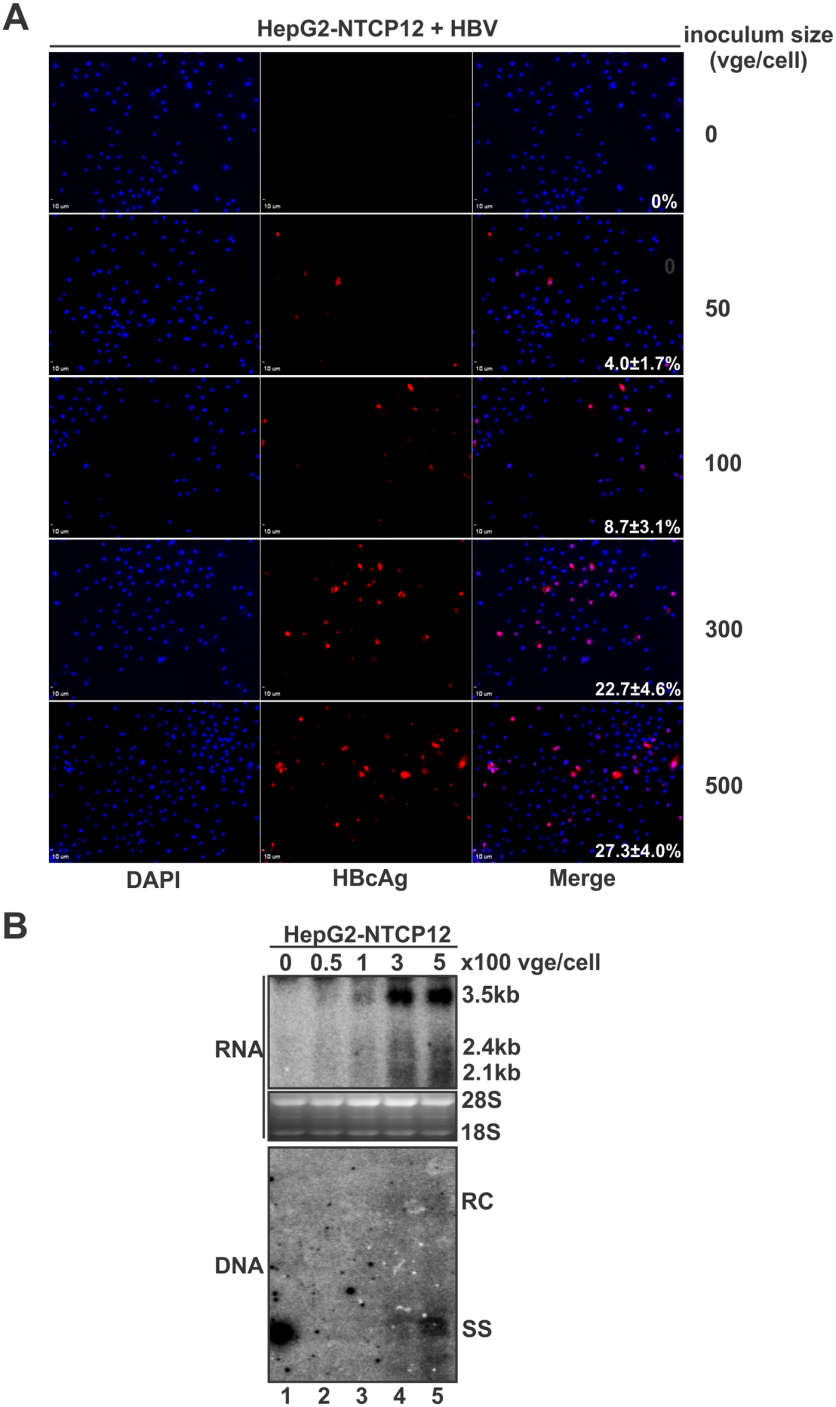
HBV infection of HepG2-NTCP12 cells with different viral inoculum size. Cells were mock infected or infected with HBV at indicated inoculum size (vge/cell) in the presence of 2% DMSO. Seven days later, the cells were subjected to HBcAg immunofluorescence microscopy (A). The intracellular HBV RNA and core DNA were analyzed by Northern and Southern blot, respectively (B).

One possible reason for the observed low infection level was the low percentage of infectious virion in the prepared HBV inoculum from cell cultures. We then performed HBV infection with different inoculum size. As shown in Fig 4, HBcAg-positive cell number and the steady state level of HBV RNA gradually raised with the increase of HBV vge per cell, and the viral core DNA became detectable by Southern blot when more than 300 vge/cell was used. There was a slight increase of HBV reproduction when compared 500 vge/cell to 300 vge/cell. Based on those observations, we chose 500 vge/cell as standard inoculum in the following experiments.

### Enhancement of HBV infection by spinoculation

**Fig 5:**
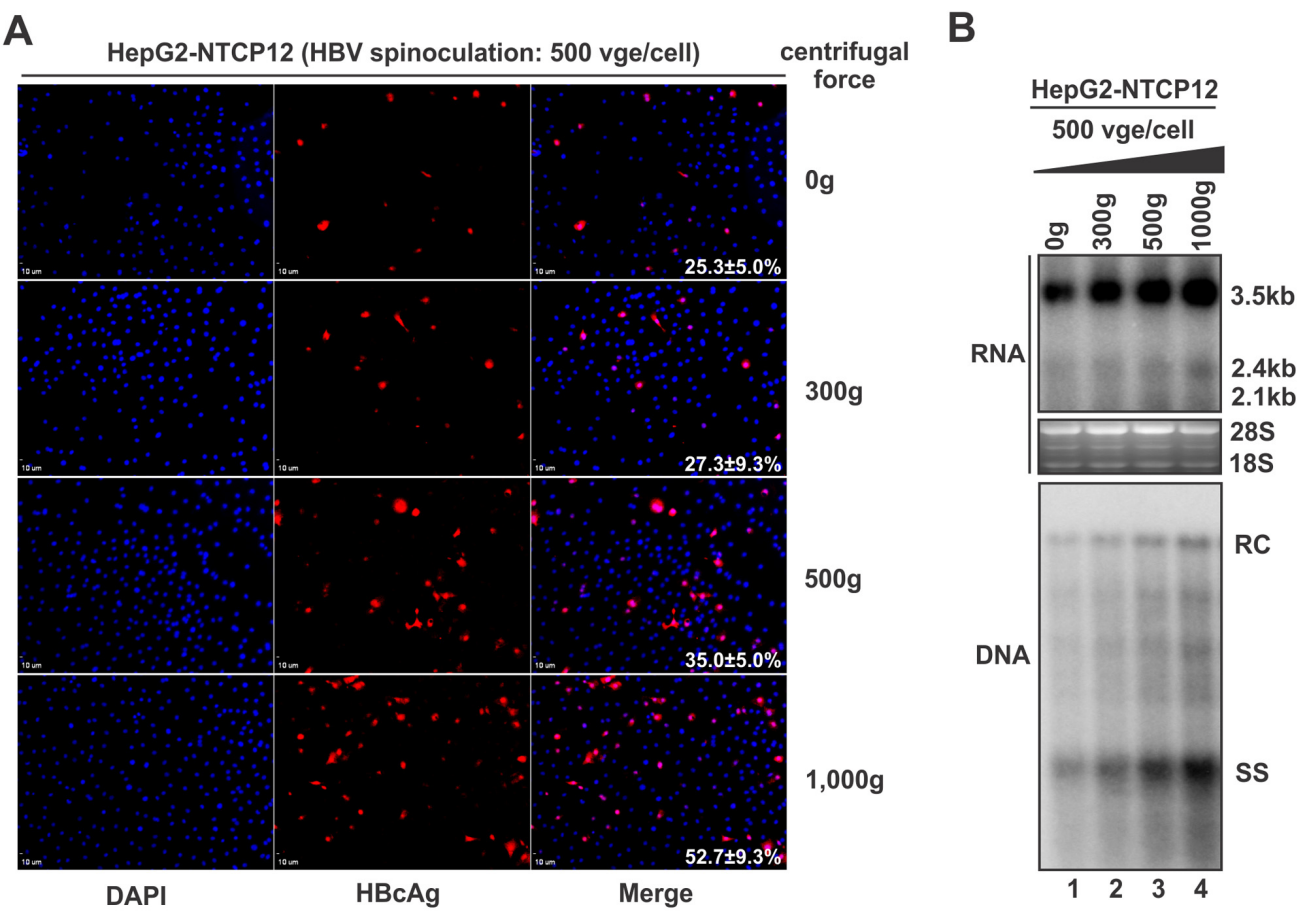
Centrifugal inoculation of HBV. HepG2-NTCP12 cells in 24-well-plate were inoculated by HBV (500 vge/cell) without centrifugation or at different centrifugal force (g) for 30 min. After spinoculation, the cells were transferred to regular culture condition. 7 days later, the infected cells were analyzed by HBcAg immunofluorescence (A), the levels of intracellular HBV RNA transcription and DNA replication were determined by Northern and Southern blot, respectively (B).

Spinoculation has been widely used to augment the infection of many different viruses, but it has not been applied in HBV infection thus far. In order to achieve a better HBV infection in HepG2-NTCP12 cells, we performed HBV infection through centrifugal inoculation. Intriguingly, HBV infection was significantly enhanced by spinoculation and positively correlated to the centrifugal force (up to 1,000g tested), as demonstrated by HBcAg immunofluorescence, HBV RNA and DNA hybridization. Spinoculation at 1,000g for 30 min enhanced HBV infection by more than two fold versus regular infection (Fig 5). No cytotoxicity or drastic cell morphological alteration was observed during the entire infection, except that the cells became noticeably flatter after centrifugation and then gradually recovered to normal morphology in the following culture period (data not shown).

**Fig 6:**
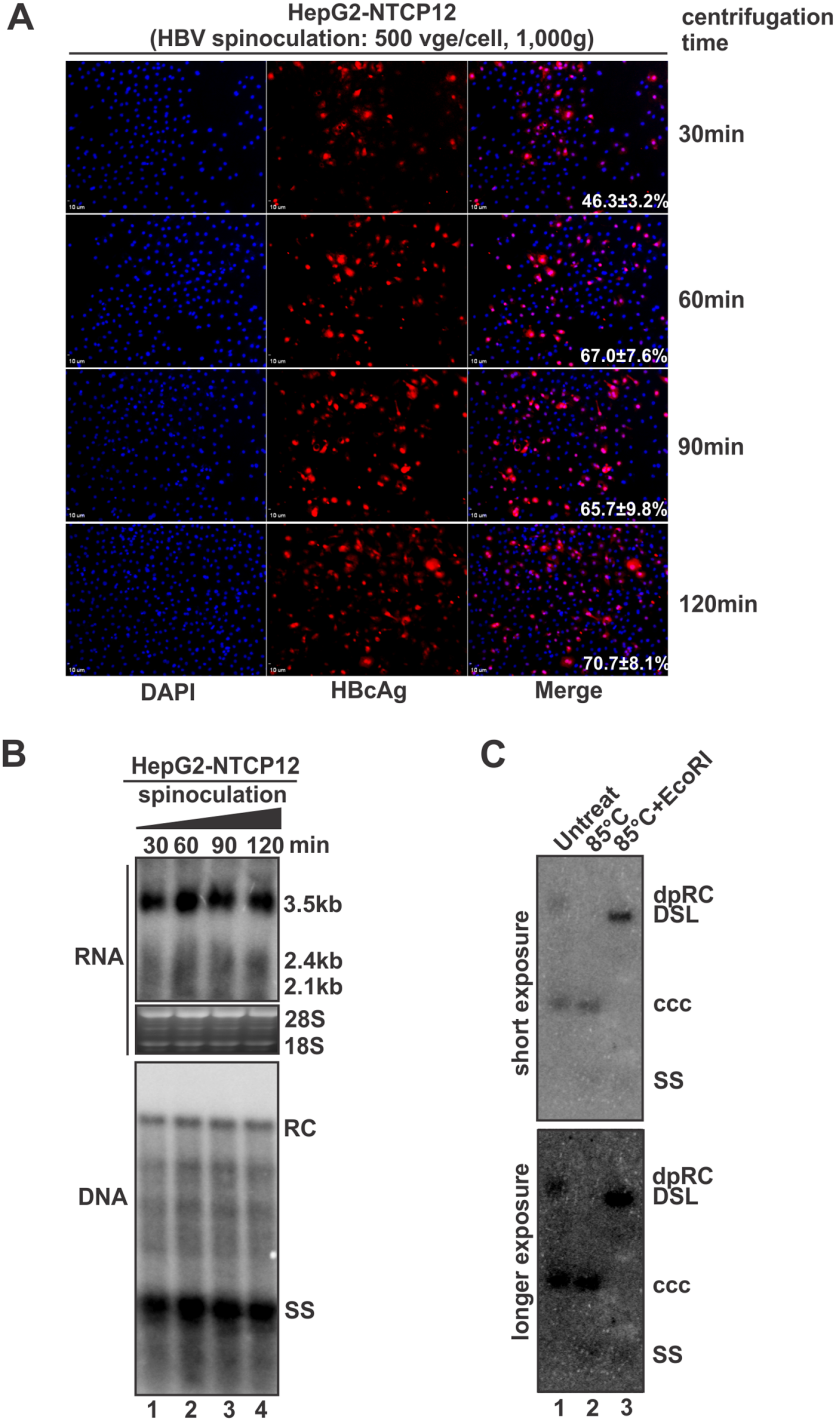
Effect of centrifugation time on HBV spinoculation. HepG2-NTCP12 cells were inoculated with HBV (500 vge/cell) by centrifugation at 1,000×g for different time as indicated. 8 days post inoculation, HBV infection was analyzed by HBcAg immunofluorescence (A), viral RNA and core DNA hybridization (B), and HBV cccDNA produced by 60-min-spinoculation was detected by Southern blot and validated by heat denature and EcoR I linearization (C).

We next assessed the effect of centrifugation time on HBV spinoculation. As shown in Fig 6A and 6B, with a fixed centrifugal force at 1,000g, extending centrifugation time from 30 min to 1 h increased the virus expression/replication by approximately 50%, but no further significant enhancement of infection was observed after prolonged centrifugation. Therefore, we finalized the HBV spinoculation condition to the following key parameters: 500 vge/cell, 1,000g, and 60 min. Under this optimized spinoculation condition, HBV cccDNA and deproteinized (protein-free) RC DNA produced in a 35-mm-dish of infected HepG2-NTCP12 cells at day 8 post infection were detected by conventional Southern blot, and the authenticity of cccDNA was validated by heat denaturing and additional EcoRI linearization according to our previously published method [25] (Fig 6C). However, HBV infection under the same above condition but without centrifugation did not produce detectable level of cccDNA by Southern blot (data not shown). The detection of cccDNA and its transcripts in the spinoculated cells clearly demonstrates that the observed HBcAg and core DNA in this system is due to infection of the cells and viral replication, and not from the detection of the HBV inoculum.

Interestingly, in contrast to HBV stable cell lines, in which the deproteinized (dp) RC DNA is an overwhelming HBV DNA species in the Hirt extraction [20, 26–28], cccDNA level was slightly more than dp-RC DNA in the Hirt DNA from HBV infected HepG2-NTCP12 cells (Fig 6C), indicating that the conversion from dp-RC DNA to cccDNA is more efficient or the level of RC DNA deproteination is low in the infected HepG2-NTCP12 cells.

### Dependence of NTCP in HBV entry during spinoculation

**Fig 7:**
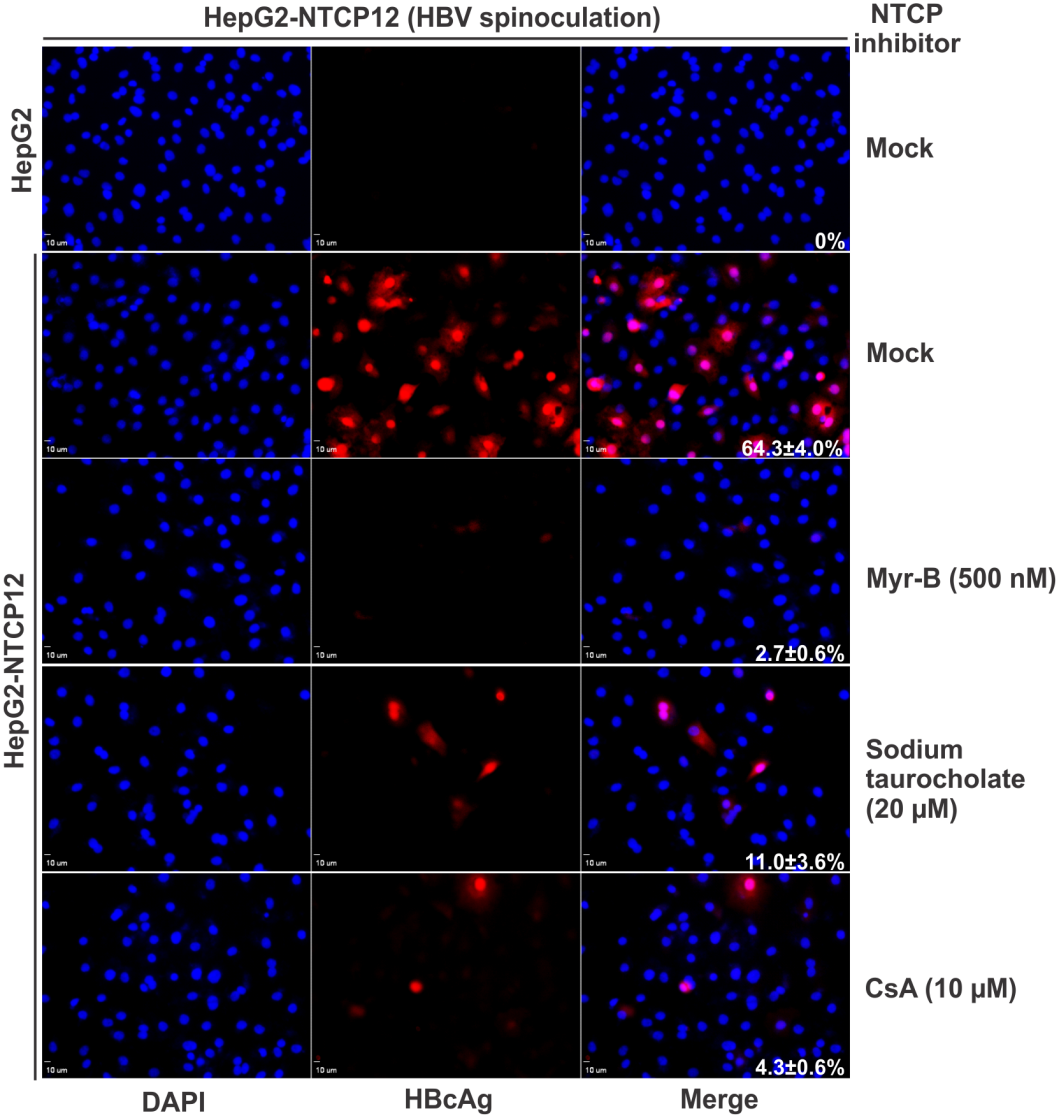
Spinoculation-mediated HBV infection is NTCP-dependent. HepG2 and HepG2-NTCP12 cells were spinoculated with HBV (500 vge/cell, 1,000×g, 60 min) in the absence or presence of indicated compounds. 8 days post inoculation, the infected cells were analyzed by HBcAg immunofluorescence.

In order to rule out a possibility that the spinoculation-mediated HBV infection might bypass the receptor NTCP and take an alternative way to invade the cells, we performed HBV spinoculation with the parental NTCP-null HepG2 cells and the HepG2-NTCP12 cells in the absence or presence of HBV entry inhibitors. As shown in Fig 7, HepG2 cells were not susceptible to HBV spinoculation (top panel); in HepG2-NTCP12 cells, spinoculation-mediated HBV infection was dramatically inhibited by HBV entry inhibitors that block the binding of HBV virion to NTCP, such as Myrcludex B (Myr-B) [9], sodium taurocholate [29], and cyclosporin A (CsA) [30, 31]. Collectively, the data suggest that spinoculation-mediated HBV infection requires virus receptor NTCP and spinoculation does not replace the authentic HBV entry route.

## Discussion

The human NTCP-complementing hepatocyte-derived cell provides a valuable tool for studying HBV infection in vitro. However, similarly to PHH and HepaRG cells, the application of this infectious system is hampered by its limited susceptibility and reliability [32, 33]. The possible explanations for the inefficient HBV infection in NTCP-reconstituted HepG2 or Huh7 cells may include, but not limited to, 1) the low infectivity of HBV inoculum. Currently, the commonly used HBV inoculum is prepared from culture supernatant of HBV producing cell lines due to their convenient accessibility. In most studies, hundreds to thousands of vge per cell were needed to inoculate the cells (Fig 4) [7, 9, 10], which indicated that the percentage of infectious virions in the cell culture-derived virus particles is low. It is thus worthwhile to screen clinical HBV-positive blood samples for highly infectious seeds to be used in cell culture experiments; 2) the potential inhibitory effect of subviral particles in HBV inoculum. The noninfectious HBV subviral particles, also known as HBsAg, contain all the three HBV envelope proteins (L/M/S) and coexist in great excess relative to the infectious virions, which may compete the binding of HBV virion to receptor(s) on the cell surface [34]; 3) the low permissiveness of recipient cells to HBV infection. To confer the susceptibility of non-permissive human hepatic cells to HBV infection, the reconstitution of NTCP is definitely a key requirement, but additional factors may also be required to support the internalization and post entry events, such as cccDNA formation and transcription. In this regard, an optimal culture condition that maintains the hepatocyte-specific cellular environment and functions is thus needed.

In order to optimize the robustness of the current HBV in vitro infection system complemented with NTCP, we developed a spinoculation method that markedly enhanced HBV infection in NTCP-expressing HepG2-NTCP12 cells compared to the regular inoculation method. Importantly, centrifugal enhancement of HBV infection remains NTCP-dependent, suggesting the authentic NTCP-mediated virus entry is boosted by spinoculation. In addition, centrifugal inoculation is easy to implement without sophisticated manipulations. To rule out a possibility that the enhancement of HBV infectivity by spinoculation is limited to this particular HepG2-NTCP12 cell line, we tested spinoculation protocol in another HepG2/NTCP cell line established recently by Seeger et al [8], and found that the HBV infection efficiency in HepG2/NTCP cells was markedly enhanced by spinoculation as well (data not shown). Therefore, spinoculation could serve as a standard procedure in the protocol for HBV infection in human hepatocyte-derived cells (HepG2 and Huh7) complemented with NTCP, and most likely in PHH and HepaRG cells as well.

Spinoculation has been used to enhance the infection of a variety of viruses, such as human and murine cytomegaloviruses [35, 36], herpes simplex virus (HSV) [37], bluetongue virus [38], adenoviruses [39], influenza A virus [40], hepatitis C virus [41], and retroviruses including HIV-1 [14, 15, 36, 42, 43]. However, the spin-mediated enhancement of virus infection is not universal, the exceptions include pseudorabies virus and Sindbis virus [13, 35]. Having been used for more than 50 years, the underlying mechanisms of spin-mediated augment of virus infection remain elusive. It is generally acknowledged that there is a simultaneous requirement for the presence of both viruses and cells during spinoculation, and the effects on both counterparts by centrifugation contribute to the enhanced infection [14, 41]. First, although the low speed (normally 100×g to 2,000×g) used in centrifugal inoculation is not powerful enough to spin down the virus particles, some studies suggested that the centrifugation may generate a concentrating effect to facilitate the contact of viruses with cell surface, removing certain rate-limiting step(s) and increasing the binding of virus to its receptor(s) [15, 42]. Such effect may be more profound in HBV spinoculation as the inoculation medium contains 4% PEG. It has been reported that HSV spinoculation bypassed the requirement of coreceptor heparan sulfate proteoglycans (HSPGs) [44], and HBV infection of HepaRG cells also utilizes hepatocyte-associated HSPGs as low affinity attachment receptors [45]. In this study, we found that HBV receptor NTCP is indispensable in spin-mediated HBV infection (Fig 7), it is therefore of interest to investigate whether spinoculation enhances the initial binding of HBV to HSPGs on cell surface or even replaces the requirement for HSPGs.

On the other hand, the centrifugal stress during spinoculation may also influence the cellular biophysical and/or biochemical behaviors, making the cells more permissive for virus infection and replication. In this regard, the mechanistic study of spinoculation-mediated HIV-1 infection revealed that centrifugation activates cofilin and subsequently triggers the dynamics of cortical actin in CD4 T cells, by which softens a major cytoskeletal barrier for HIV-1 entry and leads to a series of beneficial activities that enhance HIV-1 receptor mobilization and binding, virus entry, reverse transcription, and nuclear migration [14]. The potential centrifugal effect on actin dynamics in hepatocytes and the role of actin network in HBV and HCV infection await further investigation. However, while membrane fusion-mediated HIV-1 entry is restricted by cortical actin, it may not be a key limiting factor for HBV and HCV which infect the hepatocytes through endocytosis [46, 47]. Additionally, whether the observed enhancement of HBV infection by spinoculation is partially due to the centrifugation-mediated stimulation of HBV cccDNA formation/transcription and/or DNA replication remains unclear. It is also worthy of note that, comparing to the reported double-digit to three-figure fold enhancement of virus infection by spinoculation, the centrifugal promotion on HBV infection is only severalfold, which is similar to HCV spinoculation [41]. While there still might be a room for further optimization of HBV spinoculation, it is also possible that the hepatocytes are inherently somewhat refractory to spin-mediated enhancement of virus infection.
